# Porcine Deltacoronavirus (PDCoV) Entry into PK-15 Cells by Caveolae-Mediated Endocytosis

**DOI:** 10.3390/v14030496

**Published:** 2022-02-28

**Authors:** Shiqian Li, Dai Xiao, Yujia Zhao, Luwen Zhang, Rui Chen, Weizhe Liu, Yimin Wen, Yijie Liao, Yiping Wen, Rui Wu, Xinfeng Han, Qin Zhao, Senyan Du, Qigui Yan, Xintian Wen, Sanjie Cao, Xiaobo Huang

**Affiliations:** 1 Research Center for Swine Diseases, College of Veterinary Medicine, Sichuan Agricultural University, Chengdu 611130, China; lishiqian95@126.com (S.L.); 15838585575@163.com (D.X.); zhaoyujia2015@163.com (Y.Z.); zhlvwe@126.com (L.Z.); 18102341339@163.com (R.C.); douzo_o@163.com (W.L.); wenyimin666@163.com (Y.W.); lyj819175667@163.com (Y.L.); yueliang5189@163.com (Y.W.); wurui1977@163.com (R.W.); hanxinf@163.com (X.H.); zhao.qin@sicau.edu.cn (Q.Z.); Senyandu@163.com (S.D.); yanqigui@126.com (Q.Y.); xintian3211@126.com (X.W.); csanjie@sicau.edu.cn (S.C.); 2 Sichuan Science-Observation Experimental Station for Veterinary Drugs and Veterinary Diagnostic Technology, Ministry of Agriculture, Chengdu 611130, China; 3 National Animal Experiments Teaching Demonstration Center, Sichuan Agricultural University, Chengdu 611130, China

**Keywords:** porcine deltacoronavirus (PDCoV), clathrin-mediated endocytosis, caveolae-mediated endocytosis, macropinocytosis, cell entry

## Abstract

(1) Background: Porcine deltacoronavirus (PDCoV) is a newly emerged enteric virus affecting pig breeding industries worldwide, and its pathogenic mechanism remains unclear. (2) Methods: In this study, we preliminarily identified the endocytic pathway of PDCoV in PK-15 cells, using six chemical inhibitors (targeting clathrin-mediated endocytosis, caveolae-mediated endocytosis, macropinocytosis pathway and endosomal acidification), overexpression of dominant-negative (DN) mutants to treat PK-15 cells and proteins knockdown. (3) Results: The results revealed that PDCoV entry was not affected after treatment with chlorpromazine (CPZ), 5-(N-ethyl-N-isopropyl) amiloride (EIPA)or ammonium chloride (NH_4_Cl), indicating that the entry of PDCoV into PK-15 cells were clathrin-, micropinocytosis-, PH-independent endocytosis. Conversely, PDCoV infection was sensitive to nystatin, dynasore and methyl-β-cyclodextrin (MβCD) with reduced PDCoV internalization, indicating that entry of PDCoV into PK-15 cells was caveolae-mediated endocytosis that required dynamin and cholesterol; indirect immunofluorescence and shRNA interference further validated these results. (4) Conclusions: In conclusion, PDCoV entry into PK-15 cells depends on caveolae-mediated endocytosis, which requires cholesterol and dynamin. Our finding is the first initial identification of the endocytic pathway of PDCoV in PK-15 cells, providing a theoretical basis for an in-depth understanding of the pathogenic mechanism of PDCoV and the design of new antiviral targets.

## 1. Introduction

Porcine deltacoronavirus (PDCoV), belonging to the genus Deltacoronavirus within the family Coronaviridae, was first identified in swine in Hong Kong in 2012 [1], and can cause acute diarrhoea, vomiting, dehydration and death in new born piglets [2], sharing similar clinical symptoms with diseases caused by porcine epidemic diarrhoea virus (PEDV) and transmissible gastroenteritis virus (TGEV). In 2014, outbreaks of PDCoV were first reported in the United States [3] and were subsequently reported in Canada, China, Korea, Laos, and other countries [4,5,6,7]. Artificial inoculation with PDCoV has been proven to infect animals such as calves, chickens, and turkeys [8,9], and PDCoV is able to adapt to a variety of cell types of porcine, human and avian origin in vitro [10,11]. The PDCoV strain was recently reported for the first time in plasma samples from three Haitian children with acute undifferentiated febrile illness [12]. All of these results indicate that PDCoV has potential cross-species transmission risk and potential public health risk.

Viruses can invade host cells by membrane fusion and receptor-mediated endocytosis. Endocytosis is the process by which extracellular material enters the cell by deforming the plasma membrane. Endocytic pathways primarily include clathrin-mediated endocytosis (CME), caveolae-mediated endocytosis (CavME), clathrin- and caveolae-independent endocytosis and micropinocytosis [13]. The endocytic pathways of most coronaviruses have been identified, such as SARS-CoV, HCoV-229E, MHV, PEDV and IBV, whereas the details of some newly emerging viruses may not have been worked out, such as PDCoV and SARS-CoV-2. There are only a few literature reports about PDCoV and SARS-CoV-2 endocytosis [14,15]. Currently, the endocytic pathway of alphacoronavirus and betacoronavirus has been relatively well studied. For example, HCoV-NL63 was reported to first bind to target cells via heparan sulphate proteoglycans and interact with the ACE2 protein to trigger the recruitment of clathrin to aggregate and internalize into LLC-MK2 cells via the clathrin pathway under the action of dynamin, a process requiring both actin cytoskeleton rearrangement and an endosomal acidic environment [16]. Park, J.E. et al. [17] revealed that PEDV entry into Vero cells through the clathrin-mediated endocytic pathway is dependent on low pH and serine proteolysis using endocytic markers and inhibitors of the endocytic pathway. Wang, H.L. et al. [18] found that SARS-CoV entry into cells was dependent on low pH as well as clathrin- and caveolae-independent mechanisms. Avian infectious bronchitis virus (IBV), of the genus Gammacoronavirus, was identified to enter cells by relying on low pH and lipid rafts [19]. Wang, H. et al. [20] elucidated that IBV primarily utilizes the clathrin-mediated pathway and is subsequently transported intracellularly via early endosomes to the late endosome/lysosome pathway.

Current basic research on PDCoV is primarily focused on the exploration of viral protein structure and function [21], cellular receptors [22], apoptosis and innate immune escape [23], and antiviral studies [24,25]. However, little is known about the mechanism of PDCoV cellular entry. Some studies have shown that PDCoV can enter cells through both the endocytic pathway and the cell membrane surface pathway [26]. Trypsin promotes PDCoV replication by mediating cell-to-cell fusion transmission but is not crucial for viral entry [27] and cholesterol present in viral capsule membranes and host cell membranes can influence PDCoV invasion [28]. Therefore, we combined inhibitors and dominant-negative plasmids to address the roles of different endocytic pathways in PDCoV entry and utilized shRNA interference to further validate these results. Our study provides a theoretical basis for a deeper understanding of the pathogenesis of PDCoV and the identification of new antiviral targets.

## 2. Materials and Methods

### 2.1. Cells and Virus

Porcine kidney cells (PK-15) were maintained at 37 °C in a humidified 5% CO_2_ atmosphere in Dulbecco’s modified Eagle medium (DMEM; Gibco, Carlsbad, CA, USA) supplemented with 10% heat-inactivated foetal bovine serum (PAN-Biotech, Aigenbach, Germany) and 1% antibiotic-antimycotic (Solarbio, Beijing, China). The PDCoV strain CHN-SC2015 (GenBank accession No. MK355396) was isolated and preserved by the Research Centre for Swine Disease of Sichuan Agricultural University [2]. The PK-15 cells are cultured and preserved by the Research Centre for Swine Disease of Sichuan Agricultural University.

### 2.2. Reagents, Antibodies and Plasmids

The endocytic inhibitors used including dynasore, chlorpromazine (CPZ), methyl-β-cyclodextrin (MβCD), nystatin, ammonium chloride (NH_4_Cl), and 5-(N-ethyl-N-isopropyl) amiloride (EIPA) were purchased from Selleck Chemicals (Houston, TX, USA). Antibodies against β-actin rabbit mAb (dilution rate was 1:10,000) and anti-clathrin heavy-chain (CLTC) rabbit polyclonal (dilution rate was 1:1000) coupled with secondary goat anti-rabbit IgG antibodies were purchased from ABclonal Technology (Wuhan, China). Alexa Fluor 555-labeled donkey anti-rabbit IgG (H + L) secondary antibody was purchased from Beyotime Biotechnology (Shanghai, China). The rabbit anti-PDCoV N polyclonal antibody was generated by our laboratory (dilution rate was 1:1000) [29]. The overexpression plasmids of wild-type and dominant-negative EPS15 (DIIIΔ2 and EΔ95/295), dynamin II (GFP-Dyn-WT and GFP-Dyn-DN), and caveolin-1 (GFP-Cav-WT and GFP-Cav-DN) were kindly provided by Prof. Zhou Bin, Nanjing Agricultural University (Nanjing, China).

### 2.3. Cell Viability Assay and Inhibitor Administration

PK-15 cells were seeded into 96-well plates at a density of 3 × 10^4^ cells/well, grown for 24 h and treated with pharmacological inhibitors at the indicated concentrations for 2 h. After two washes with PBS, 10 μL of cell counting kit-8 (CCK-8) solution were added to 90 μL of DMEM in each well and incubated at 37 °C for 1 h, and then the absorbance at 450 nm was measured using a microplate reader. The experiments were independently repeated three times. Confluent monolayers of PK-15 cells were grown in 6-well plates and pre-treated with the appropriate concentration of inhibitor for 1 h. Then, the cells were infected with PDCoV (MOI = 0.1) in the presence of drugs for 1 h at 37 °C. After washing with PBS, the cells were incubated with medium containing trypsin for 24 h and subsequently collected for qRT-PCR and Western blotting analysis.

### 2.4. Binding and Entry Assay

PK-15 cells were seeded into 6-well plates, grown for 24 h and treated with pharmacological inhibitors at the indicated concentrations for 37 °C 1 h. Then, they were infected with PDCoV (MOI of 5) at 4 °C for 1 h (binding step), and then shifted to 37 °C for 1 h (entry step). Cells were lysed to determine viral mRNA levels by qRT-PCR.

### 2.5. Plasmid Construction and Lentivirus Production

The three pairs of shRNA sequences targeting Sus scrofa clathrin heavy chain (CLTC), caveolin-1 (CAV1) and dynamin-2 (DNM2) were listed in Table S1 and were cloned into pLenti-CMV-V2-puro to generate the pLenti-CMV-shCLTC, pLenti-CMV-shCAV1 and pLenti-CMV-shDNM2, respectively. The construct plasmids with two other plasmids (psPAX2, pMD2.G) were co-transfected into HEK293T cells using Lipofectamine 3000 Transfection Reagent (Thermo Fisher Scientific, Waltham, MA, USA). Supernatants containing the lentivirus were collected at 36 h and 48 h after transfection, respectively. The lentivirus was applied to infect PK-15 cells. After 36 h infection, PK-15 cells were cultured in fresh medium with puromycin (5 ug/mL) to select stable cell lines.

### 2.6. qRT-PCR and Western Blotting

The expression of PDCoV N protein was assessed by Western blotting and the mRNA levels of PDCoV N gene were detected by qRT-PCR with β-actin as the reference. Total RNA was extracted using a UNlQ-10 Column TRIzol Total RNA Isolation Kit (Sangon, Shanghai, China), and then subjected to qRT-PCR using primers specific to the N gene of PDCoV. Primer sequences used for qRT-PCR were listed in Table S2. SYBR Green I Master Mix (TaKaRa, Dalian, China) was used for the qRT-PCR reactions and the amplification conditions were 95 °C for 30 s, then 40 cycles of 95 °C for 5 s, 55 °C for 30 s, and 72 °C for 30 s using the LightCycler 96 system (Roche, Mannheim, Germany). For the Western blotting analysis, the cells were washed with PBS and lysed in RIPA lysis buffer and PMSF on ice for 20 min. After SDS-PAGE, proteins were transferred onto PVDF membranes. The membranes were blocked with 5% skim milk in PBST (PBS/0.05% Tween-20) for 1.5 h and then incubated with the corresponding antibodies at 4 °C overnight. Membranes were washed four times with PBST and then incubated with HRP-goat anti-Rabbit IgG (1:5000) for 1 h at 37 °C. The membrane was washed four times with PBST and the proteins were visualized using enhanced chemiluminescence reagents (ECL; Bio–Rad, Hercules, CA, USA).

### 2.7. Indirect Immunofluorescence Assay

PK-15 cells seeded in 12-well plates with coverslips were left untreated or pretreated with indicated inhibitors for 1 h, and incubated with 10 μg/mL Tfn for 30 min at 4 °C and then transferred to 37 °C for 30 min. Then, cells were washed with cold PBS three times, fixed with 4% paraformaldehyde. Cell nuclei were stained with DAPI and observed by fluorescence microscopy.

PK-15 cells were grown to 50% confluence on coverslip dishes and transfected with 2.5 μg of the indicated plasmids using Lipofectamine 3000, according to the manufacturer’s instructions. After 24 h, the cells were incubated with Tfn at 10 ug/mL for 30 min at 4°C and then transferred to 37 °C for 30 min or infected with PDCoV. At 24 hpi, cells were fixed in 4% paraformaldehyde for 30 min, permeabilized with 0.2% Triton X-100 for 30 min, blocked with 5% bovine serum albumin (BSA) for 1 h at 37 °C, incubated with rabbit anti-PDCoV N polyclonal antibody (1:200) at 4 °C overnight and Alexa Fluor 555-labelled donkey anti-rabbit IgG (H + L) (1:500) for 1 h at room temperature. Cell nuclei were stained with DAPI and observed by fluorescence microscopy.

### 2.8. Statistical Analysis

All graphs were created using GraphPad Prism 8 software. All data are presented as the means ± standard deviations (SDs) from at least three independent experiments. Significance was estimated using two-tailed Student’s *t*-tests. *p*-values less than 0.05 were defined as the threshold for statistical significance. *p*-values between 0.05 and 0.01 are designated by one asterisk, *p*-values between 0.01 and 0.001 are designated by two asterisks, *p*-values between 0.001 and 0.0001 are designated by three asterisks, and *p*-values less than 0.0001 are designated by four asterisks.

## 3. Results

### 3.1. Cell Viability Assay

To determine optimal working concentrations, the cytotoxicity of the inhibitors used in the experiments were determined using a Cell Counting Kit-8 (CCK-8) kit. According to the results shown in Figure S1, suitable concentrations of the inhibitors were as follows: chlorpromazine (CPZ): 5, 10, 20 (μM); Dynasore: 40, 80, 100 (μM); ammonium chloride (NH_4_Cl): 5, 10, 20 (mM); nystatin: 5, 10, 20 (μM); 5-(N-ethyl-N-isopropyl) amiloride (EIPA): 10, 20,30 (μM); methyl-β-cyclodextrin (MβCD): 0.1, 0.5, 1 (mM). For all of these concentrations, the cell viability was greater than 90%, and there were no effects on cell morphology.

### 3.2. PDCoV Entry into PK-15 Cells Does Not Depend on the Clathrin-Mediated Endocytosis Pathway

**Figure 1:**
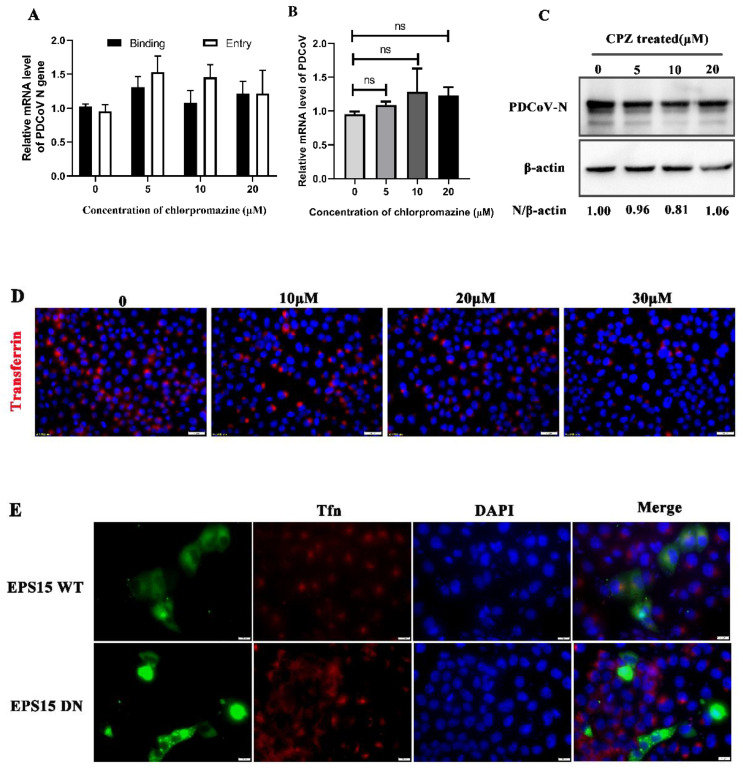

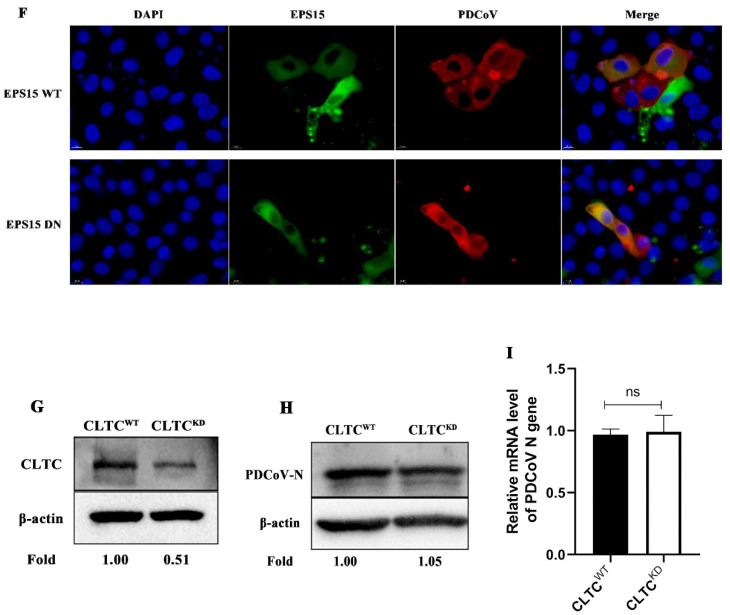
PDCoV entry into porcine kidney (PK-15) cells does not depend on the clathrin-mediated endocytosis pathway (**A**). PK-15 cells were pretreated with chlorpromazine (CPZ) at 37 °C for 1 h and infected with PDCoV (MOI of 5) at 4 °C for 1 h (binding step), and then shifted to 37 °C for 1 h (entry step). Cells were lysed to determine viral mRNA level by qRT-PCR. (**B**,**C**). Cells were mock-treated or treated with CPZ for 1 h followed by infection with PDCoV (MOI = 0.1). At 24 hpi, mRNA expression levels of total PDCoV were detected by RT-qPCR (**B**) and protein expression levels of intracellular PDCoV were detected by Western blotting (**C**). (**D**) PK-15 cells were pretreated with CPZ with different concentrations at 37 °C for 1 h, followed by incubation with 10 μg/mL Alexa Fluor 568-labeled transferrin (Tfn) on ice for 30 min, then shifted to 37 °C for 30 min. The cells were fixed and stained with DAPI (Scale bar, 20 μm). (**E**,**F**) PK-15 cells were transfected with EPS15 wild type (WT) or EPS15 dominant-negative (DN), followed by incubation with 10 μg/mL Tfn or infection with PDCoV (MOI = 0.1). At 24 hpi, the cells were fixed and immunostained (Scale bar, 10 μm). (**G**–**I**) Clathrin heavy-chain knockdown cells and normal cells were infected with PDCoV (MOI = 0.1). Knockdown efficiency was verified with Western blotting (**G**). At 24 hpi, the viral N protein and viral RNA levels were determined by Western blotting (**H**) and qRT-PCR (**I**), respectively. ns: there was no significant difference.

Clathrin-mediated endocytosis (CME) represents a classical endocytosis pathway. Most viruses use this pathway to enter cells. To determine whether PDCoV can gain entry into PK-15 cells through this pathway, we treated the cells with chlorpromazine, an inhibitor of the CME pathway, which inhibits the CME pathway by blocking the formation of clathrin and AP2 complexes at the plasma membrane; thereby, chlorpromazine inhibits the assembly of clathrin-coated pits [30]. As shown in Figure 1D, transferrin (Tfn) uptake was significantly reduced but chlorpromazine had no effect on PDCoV binding and entry (Figure 1A). After PDCoV internalization, there were also no significant differences on PDCoV protein and mRNA expression levels compared with the control group (Figure 1B,C). EPS15 presents at clathrin-coated pits and involved in receptor-mediated endocytosis [31]. Besides, it is necessary for proper coated vesicle formation, so we sought to confirm whether EPS15 is involved in PDCoV entry. Then, the wild-type (WT) and dominant-negative mutant plasmids (DN) of EPS15 were transfected into cells, followed by incubation with transferrin or infection with PDCoV 24 h later. Immunofluorescence revealed that EPS15 DN inhibited Tfn uptake (Figure 1E) and both groups were colocalized with PDCoV (Figure 1F). To further confirm these results, lentivirus packaging to interfere with the expression of clathrin heavy chain (CLTC) was performed and protein knockdown efficiency was detected by Western blotting (Figure 1G). According to Figure 1H,I, no significant differences in PDCoV N protein and mRNA expression levels were found. These results all suggest that PDCoV entry into PK-15 cells is independent of the clathrin-mediated endocytosis pathway.

### 3.3. PDCoV Entry into PK-15 Cells Depends on the Caveolae-Mediated Endocytosis Pathway

**Figure 2:**
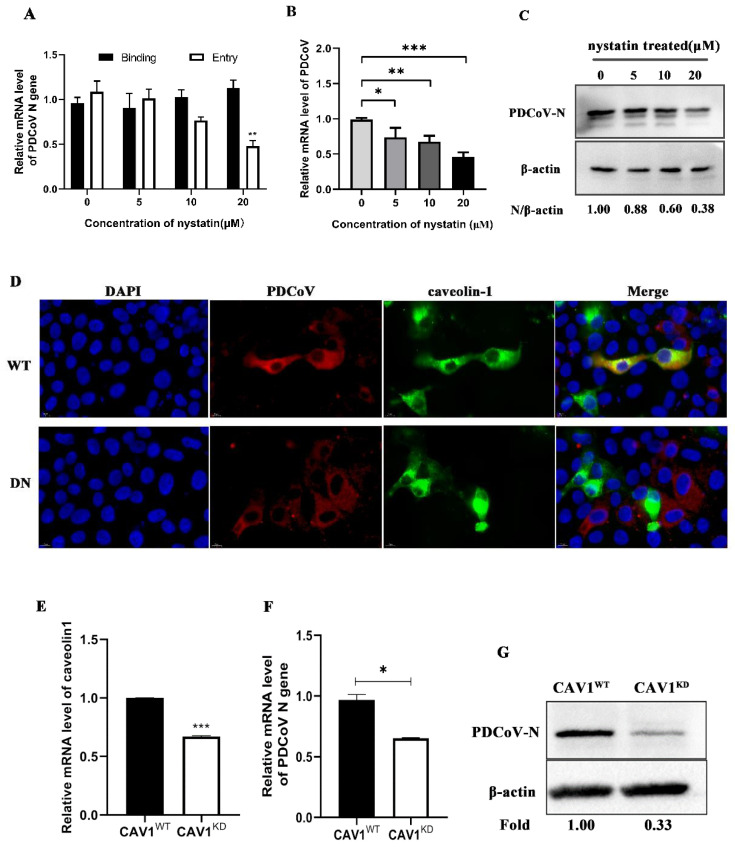
PDCoV entry into porcine kidney (PK-15) cells depends on the caveolae-mediated endocytosis pathway. (**A**). PK-15 cells were pretreated with nystatin at 37 °C for 1 h and infected with PDCoV (MOI of 5) at 4 °C for 1 h (binding step), and then shifted to 37 °C for 1 h (entry step). Cells were lysed to determine viral mRNA level by qRT-PCR (**B**,**C**). Cells were mock-treated or treated with nystatin for 1 h followed by infection with PDCoV (MOI = 0.1). At 24 hpi, mRNA expression levels of total PDCoV were detected by real-time quantitative PCR (**B**) and protein expression levels of intracellular PDCoV were detected by Western blotting (**C**). (**D**) PK-15 cells were transfected with caveolin-1 wild type (WT) or caveolin-1 dominant-negative (DN), followed by infection with PDCoV 24 h later (MOI = 0.1). At 24 hpi, the cells were fixed and immunostained. Scale bar, 10 μm. (**E**–**G**) Caveolin-1 knockdown and normal cells were infected with PDCoV (MOI = 0.1). The knockdown efficiency was verified by qRT-PCR (**E**). At 24 hpi, the viral RNA and N protein levels were determined by qRT-PCR (**F**) and Western blotting (**G**), respectively. * *p* < 0.05, ** *p* < 0.01, *** *p* < 0.001.

To determine whether PDCoV entry into PK-15 cells occurs through the caveolae-mediated pathway (CavME), nystatin (a specific inhibitor of the CavME) was used. To determine the effect of nystatin on PDCoV entry and infection, qRT-PCR and Western blotting were performed. As shown in Figure 2A, nystatin inhibited PDCoV entry but did not inhibit PDCoV binding, and the mRNA level of PDCoV decreased in a dose-dependent manner. According to Figure 2B, the replication of PDCoV decreased significantly with increasing inhibitor concentration and was decreased by 0.45-fold at the highest concentration. As shown in Figure 2C, the expression levels of the PDCoV N protein in PK-15 cells were decreased in a dose-dependent manner and were decreased to 38% of control levels in response to 20 μM. Next, the wild-type or mutant plasmids of caveolin-1 (the primary structural protein and regulatory component of caveolae) were transfected into cells. The results showed that WT plasmids colocalized with PDCoV, while the DN group did not (Figure 2D). To further confirm our conclusion, the expression of caveolin-1 was knocked down (Figure 2E). qRT-PCR was performed to detect protein knockdown efficiency because no specific band could be detected by Western blotting, which may be due to antibody homology. Although the mRNA level of caveolin-1 decreased by about 45% in the knockdown cells, PDCoV infection was still significantly reduced by 35% and 67%, respectively, in Figure 2F,G. These results suggest that cellular entry of PDCoV depends on the caveolae-mediated endocytosis pathway.

### 3.4. PDCoV Entry into PK-15 Cells Relies on Cholesterol

**Figure 3:**
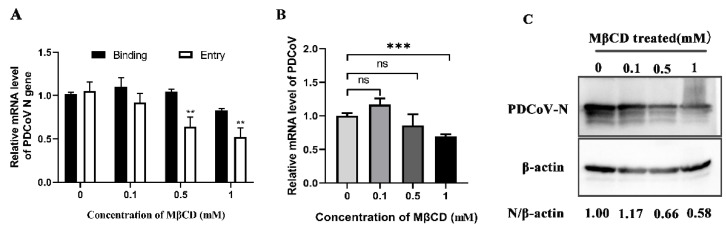
PDCoV entry into PK-15 cells relies on cholesterol. (**A**) PK-15 cells were pretreated with methyl-β-cyclodextrin (MβCD) at 37 °C for 1 h and infected with PDCoV (MOI of 5) at 4 °C for 1 h (binding step), and then shifted to 37 °C for 1 h (entry step). Cells were lysed to determine viral mRNA level by RT-qPCR. (**B**,**C**). Cells were mock-treated or treated with MβCD for 1 h followed by infection with PDCoV (MOI = 0.1). At 24 hpi, mRNA expression levels of total PDCoV were detected by real-time quantitative PCR (**B**) and protein expression levels of intracellular PDCoV were detected by Western blotting (**C**). ** *p* < 0.01, *** *p* < 0.001.

Cholesterol plays an important role in the life cycle of the virus and many coronaviruses need to have cholesterol on the viral capsule or cell membrane to enter cells [32,33]. To determine whether PDCoV infection is cholesterol-dependent, the cholesterol extractant–MβCD was used. As shown in Figure 3A, MβCD had no effect on PDCoV binding but inhibited PDCoV entry. After 24 hpi, there was no significant effect on the expression level of PDCoV N protein at 0.1 mM (the lowest concentration of MβCD), while PDCoV replication was significantly reduced to 66% at 0.5 mM and 58% at 1 mM; qRT–PCR results were consistent with the trend observed in Western blotting (Figure 3B,C). All these results indicate that MβCD inhibits PDCoV internalization and cholesterol is required for PDCoV infection.

### 3.5. PDCoV Cellular Entry Depends on Dynamin

**Figure 4:**
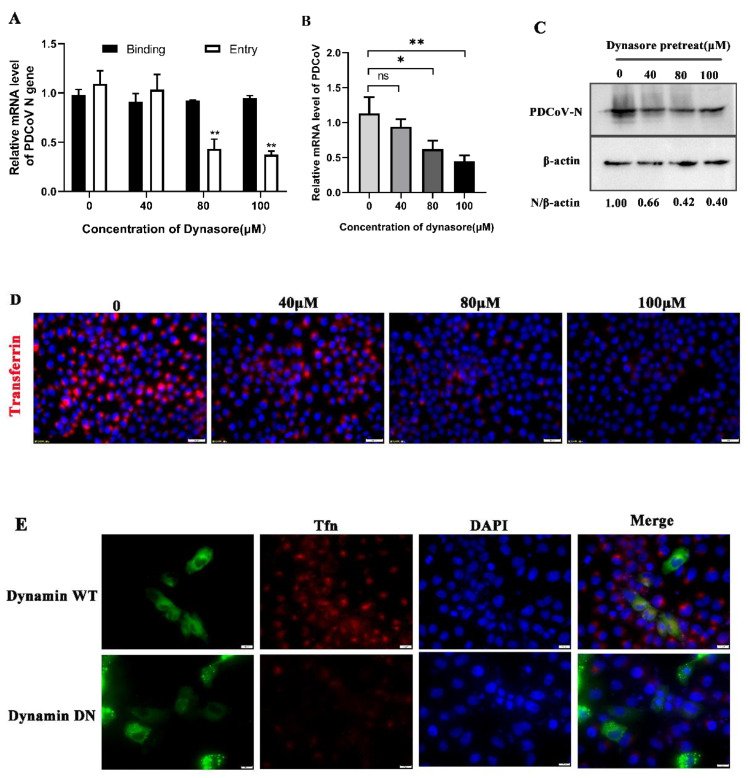

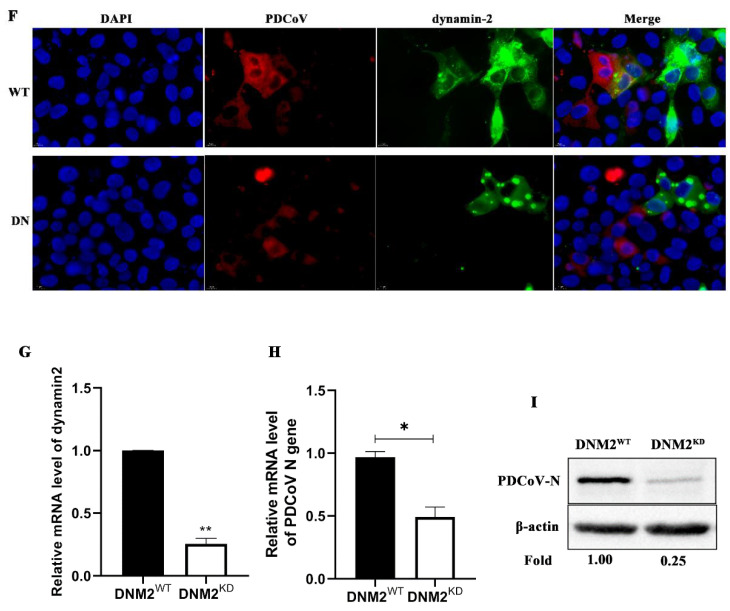
PDCoV entry depends on dynamin. (**A**) PK-15 cells were pretreated with dynasore at 37 °C for 1 h and infected with PDCoV (MOI of 5) at 4 °C for 1 h (binding step), and then shifted to 37 °C for 1 h (entry step). Cells were lysed to determine viral mRNA level by RT-qPCR.Cells were mock-treated or treated with dynasore for 1 h followed by infection with PDCoV (MOI = 0.1). At 24 hpi, mRNA expression levels of total PDCoV were detected by real-time quantitative PCR (**B**) and protein expression levels of intracellular PDCoV were detected by Western blotting (**C**). (**D**) PK-15 cells were pretreated with dynasore with different concentrations at 37 °C for 1 h, followed by incubation with 10 μg/mL Alexa Fluor 568-labeled transferrin (Tfn) on ice for 30 min, then shifted to 37 °C for 30 min. The cells were fixed and stained with DAPI. Scale bar, 20 μm. (**E**,**F**) PK-15 cells were transfected with dynamin-2 wild type (WT) or dynamin-2 dominant-negative (DN), followed by incubation with 10 μg/mL Tfn or infection with PDCoV 24 h later (MOI = 0.1). At 24 hpi, the cells were fixed and immunostained. Scale bar, 10 μm. (**G**–**I**) Dynamin-2 knockdown and normal cells were infected with PDCoV (MOI = 0.1). The knockdown efficiency was verified by qRT-PCR (**G**). At 24 hpi, the viral RNA and N protein levels were determined by qRT-PCR (**H**) and Western blotting (**I**), respectively. * *p* < 0.05, ** *p* < 0.01, ns: no significant difference.

Next, to determine whether PDCoV entry depends on dynamin, we treated PK-15 cells with dynasore, an inhibitor of dynamin, and subsequently infected them with PDCoV. As shown in Figure 4A,D, Tfn uptake and PDCoV entry was significantly reduced after dynasore treatment but PDCoV binding was not inhibited. At 24 hpi, the protein and mRNA expression levels of PDCoV were also significantly reduced in a dose-dependent manner (Figure 4B,C). Viral replication was decreased to 66%, 42% and 40% at 40 μM, 80 μM and 100 μM, respectively (Figure 4C). However, in Figure 4B, there was a significant difference compared to the control group only at 80 μM and 100 μM (the higher concentrations). Furthermore, the wild-type or mutant plasmids of dynamin-2 were transfected into cells, followed by incubation with Tfn or infection with PDCoV 24 h later. Immunofluorescence revealed that there was no colocalization with Tfn in DN positive cells but WT did (Figure 4E). WT plasmids also colocalized with PDCoV, while the DN group did not, indicating that dynamin is required for PDCoV entry (Figure 4F). To further investigate the role of dynamin in PDCoV entry into PK-15 cells, the expression of dynamin-2 was knocked down by utilizing lentivirus packaging. qRT-PCR was performed to detect the mRNA level of dynamin-2. The mRNA level of dynamin-2 was decreased by about 80% in DNM2 knockdown cells (Figure 4G). At 24 hpi, viral infection was evaluated using qRT-PCR and Western blot. PDCoV infection was significantly reduced in dynamin-2 knockdown cells (Figure 4H,I). Collectively, these results demonstrate that PDCoV cellular entry requires dynamin.

### 3.6. PDCoV Entry Does Not Depend on Macropinocytosis

**Figure 5:**
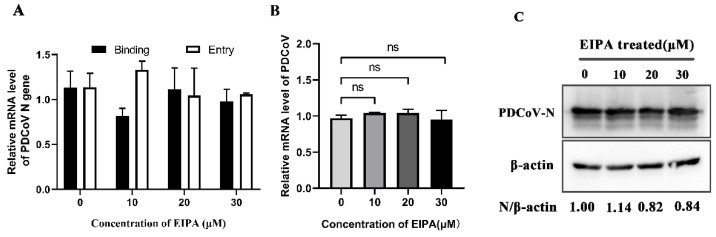
PDCoV entry does not depend on macropinocytosis. (**A**) PK-15 cells were pretreated with 5-(N-ethyl-N-isopropyl) amiloride (EIPA) at 37 °C for 1 h and infected with PDCoV (MOI of 5) at 4 °C for 1 h (binding step), and then shifted to 37 °C for 1 h (entry step). Cells were lysed to determine viral mRNA level by RT-qPCR. Cells were mock-treated or treated with EIPA for 1 h followed by infection with PDCoV (MOI = 0.1). At 24 hpi, mRNA expression levels of total PDCoV were detected by real-time quantitative PCR (**B**) and protein expression levels of intracellular PDCoV were detected by Western blotting (**C**). ns: no significant difference.

To verify whether PDCoV enters PK-15 cells through macropinocytosis, we treated PK-15 cells with EIPA, an inhibitor of the macropinocytosis pathway. Our data revealed that EIPA had no effect on PDCoV binding and entry (Figure 5A). At 24 hpi, with increasing drug concentration, there was also no significant difference in mRNA levels of related genes compared to the control group (Figure 5B) or the expression levels of PDCoV N protein (Figure 5C), indicating that PDCoV entry into PK-15 cells does not depend on the macropinocytic pathway.

### 3.7. PDCoV Enters PK-15 Cells Is Low-pH-Independent Manner

**Figure 6:**
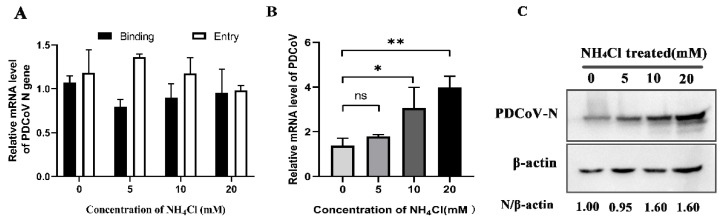
PDCoV enters PK-15 cells in a low-pH-independent manner. (**A**) PK-15 cells were pretreated with ammonium chloride (NH_4_Cl) at 37 °C for 1 h and infected with PDCoV (MOI of 5) at 4 °C for 1 h (binding step), and then shifted to 37 °C for 1 h (entry step). Cells were lysed to determine viral mRNA level by RT-qPCR. Cells were mock-treated or treated with NH_4_Cl for 1 h followed by infection with PDCoV (MOI = 0.1). At 24 hpi, mRNA expression levels of total PDCoV were detected by real-time quantitative PCR (**B**), and protein expression levels of intracellular PDCoV were detected by Western blotting (**C**). * *p* < 0.05, ** *p* < 0.01, ns: no significant difference.

To determine whether PDCoV is pH-dependent, we treated PK-15 cells with ammonium chloride (NH_4_Cl), an inhibitor of acidification. After infection with PDCoV, the protein and mRNA expression levels of PDCoV were detected by Western blot and qRT-PCR. According to the results in Figure 6C, N protein expression levels of PDCoV were increased in response to treatment with different concentrations of ammonium chloride and increased by 1.6-fold in response to 10 mM and 20 mM. qRT-PCR results (Figure 6B) also demonstrated that mRNA levels were increased with increasing drug concentration, and there was a significant difference between 10 mM and 20 mM (*p* < 0.05). However, there were no obvious effects on PDCoV binding and entry (Figure 6A). These results verify that PDCoV entry into PK-15 cells does not depend on the acidic environment of endosomes.

## 4. Discussion

PDCoV has become an important pathogen causing porcine diarrhoeal disease in the pig industry worldwide. The mechanism by which PDCoV infects host cells is not completely clear, and there are currently few related studies on endocytosis. Therefore, performing endocytosis-related studies is of great significance for understanding the pathogenic mechanism of PDCoV. This study is the first initial characterization of the endocytic pathway and intracellular trafficking of PDCoV on PK-15 cells, and it is important to gain new insight into the pathogenic mechanism and antiviral target design of PDCoV.

The clathrin-mediated endocytic pathway (CME) is a classical endocytic pathway by which enveloped viruses enter cells [34], such as MHV-2 virus entry into mouse astrocytoma cells [35], porcine haemagglutinating encephalomyelitis virus (PHEV) entry into mouse neuroblastoma cells (Neuro-2a) [36] and others. In this study, whether PDCoV also utilizes this classical pathway to enter cells was examined using chemical inhibitors, the construction of EPS 15 dominant-negative cells and lentivirus packaging. Chlorpromazine inhibits the CME pathway by blocking the formation of clathrin and AP2 complexes at the plasma membrane, thereby inhibiting the assembly of clathrin-coated pits [30]. Combined with chlorpromazine inhibition and clathrin heavy-chain knockdown, we found that PDCoV entering cells did not depend on the CME pathway. EPS15 is a key regulatory protein of the CME pathway. However, MHV-2 virus entry into mouse astrocytoma cells is via a clathrin-mediated endocytic pathway independent of EPS15 [35]. Our data showed that PDCoV infection also did not depend on EPS15. These results suggested that PDCoV entered PK-15 cells via the clathrin-independent endocytic pathway.

The caveolae-mediated endocytic pathway is another important route of viral entry that has been gradually uncovered in recent years in which caveolin-1 is a major structural protein and regulatory component of caveolae [37]. In this study, nystatin (a sterol binder that breaks down caveolae), a dominant-negative form of caveolin-1 and caveolin-1 knockdown cells were used to inhibit this pathway. The results showed that different concentrations of nystatin inhibited PDCoV internalization, and PDCoV infection was reduced in caveolin-1 DN mutant transfection cells as well as caveolin-1 knockdown cells, suggesting that PDCoV entered cells through the caveolae-mediated pathway. Cholesterol is an important component of caveolae’s structure and its absence prevents the cell membrane from forming a pit [38]. In addition, many coronaviruses need to have cholesterol on the viral capsule or cell membrane to enter cells [32,33], so cholesterol plays an important role in the life cycle of the virus. Therefore, we used MβCD to determine whether cholesterol has an effect on PDCoV entry. The results showed that MβCD significantly inhibit PDCoV entry but made no difference on binding. Our results are consistent with that reported by Jeon, J.H. et al. [28]. Although they identified this phenomenon, there was no in-depth study of the mechanism of cholesterol’s action. In our study, viral infection declined only at higher concentrations of MβCD, possibly because low concentrations of MβCD did not adequately antagonize cholesterol in the cell membrane and viral capsule. Based on the results of this study, we speculate that cholesterol affects the caveolae-mediated endocytosis pathway by affecting caveolae’s structural integrity, thus affecting the entry of PDCoV. Dynamin is a GTPase, which plays a dual role in CME: It acts as a fidelity monitor to regulate clathrin-coated pit maturation in the early stage and directly catalyzes membrane fission and clathrin-coated vesicle formation in the late stage [39]. Besides, dynamin is involved in the internalization of the caveolae. It contributes to the release of the caveolin by hydrolyzing GTP and lack of dynamin prevents vesicle division. Therefore, to determine whether PDCoV enter cells depends on dynamin, dynasore inhibition, dynamin DN mutant transfection and dynamin knockdown were performed. The results revealed that dynamin was required for the PDCoV life cycle including entry and replication. In addition to CME and CavME, the virus can also enter cells via the macrocytosis pathway, which is a nonspecific endocytosis pathway [40]. We next used EIPA to treat PK-15 cells and found that PDCoV internalization was not affected, indicating that PDCoV entry did not depend on micropinocytosis.

Endocytic transport is influenced by the intracellular pH environment and is regulated by a series of small G proteins of the Rab family. Membrane fusion of coronavirus is generally considered to occur under neutral pH conditions [19], but some coronaviruses’ entry into cells relies on the acidic environment of endosomes, such as IBV [19] and SARS [16]. The intracellular transport process is gradually acidic and lysosomes are the terminal organelles on the endocytic pathway [41]. When inhibitors of acidification are used, the intracellular pH increases, and the function of lysosome hydrolysis is blocked, resulting in the vesicles being unable to release the virus [42,43]. In this study, PDCoV infection was increased after using NH_4_Cl and we speculated that virus accumulated in the endocytosis vesicles, resulting in the increase of intracellular virus. Besides, it has been shown that PDCoV can enter cells through endocytosis and the cell membrane surface pathway, so it is not sufficient to inhibit viral entry using nucleosome inhibitors alone [24]. Therefore, we also speculate that the endocytosis pathway is inhibited in response to ammonium chloride but that it provides favourable conditions for the cell membrane surface pathway that promotes viral entry into cells.

So far, no more studies about PDCoV endocytosis have been reported, except for Fang, P. et al. [14]. They revealed that PDCoV (CHN-HN-2014) enters IPI-2I cells via macropinocytosis and clathrin-mediated endocytosis that requires a low-pH environment and dynamin, while PDCoV enters ST cells and LLC-PK1 cells via the caveola-mediated endocytic pathway [14]. Due to the multidirectional regulation of endocytosis and cell-type dependence, virus may enter the cell in one or more ways, and there may be differences among different cells and virus strains [44,45]. Therefore, we will carry out related studies on PDCoV entry in different cell lines in the future.

## 5. Conclusions

We conducted a preliminary study to explore the mechanism of PDCoV entry into PK-15 cells. Our data suggest that PDCoV entry into PK-15 cells occurs through caveolae-mediated endocytosis and is dependent on dynamin and cholesterol. Our study provides a new insight into the invasion mechanism of PDCoV and will be helpful for antiviral target design.

## Supplementary Materials

The following are available online at https://www.mdpi.com/article/10.3390/v14030496/s1, Figure S1: Cell viabilities in response to inhibitor treatment. Table S1: Sequence of shRNA used to lentivirus packaging. Table S2: Primers used for real-time RT-PCR assay.
