# Supplementary material for: Porcine Deltacoronavirus (PDCoV) Entry into PK-15 Cells by Caveolae-Mediated Endocytosis
Source: Viruses. 2022 Feb 28;14(3):496. doi: 10.3390/v14030496 (PMC8950576; doi:10.3390/v14030496)
Supplement: Supplementary file 1 [file viruses-14-00496-s001.zip › viruses-1580955-supplementar.pdf]

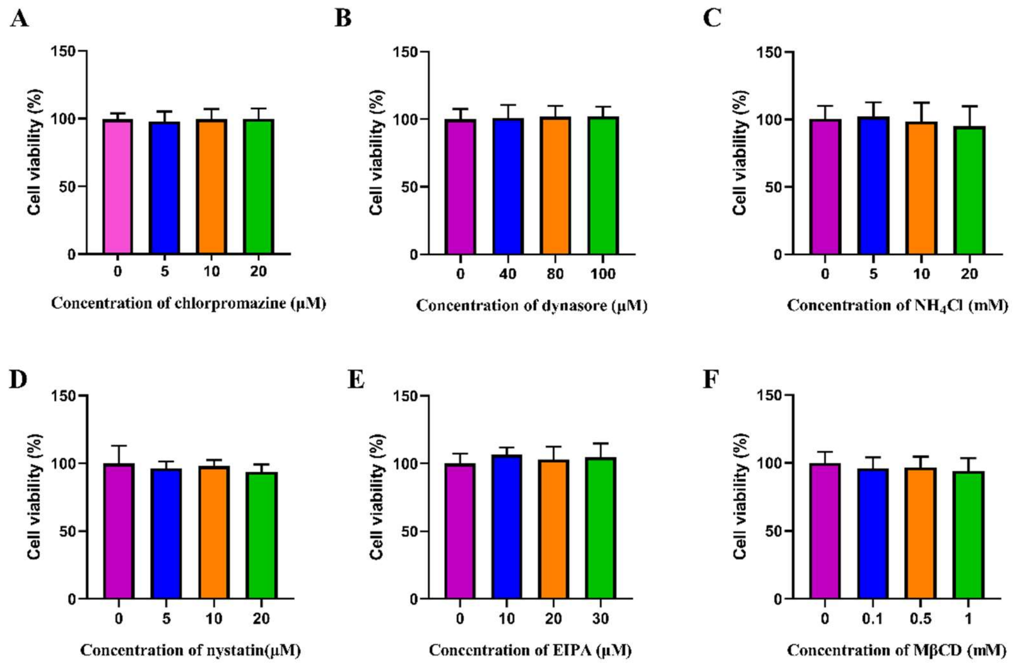

Figure S1: Cell viabilities in response to inhibitor treatment.

Table S1. Sequence of shRNA used to lentivirus packaging

**shRNA**	**Target sequence (5'→3')**
shCLTC	ATGCGTATCAGTCCAGATCA
shCAV1	AGTGTATGACGCGCACACCA
shDNM2	GTTCAGGAATCGTCACTCGG

Table S2. Primers used for real-time RT-PCR assay

**Primer**	**Nucleotide sequence (5'→3')**
Caveolin 1-F	GATCCCAAGCATCTCAACGA
Caveolin 1-R	AGAGGGCAGACAGCAAACG
dynamin2-F	TCCCACTCGCAAGACCAAA
dynamin2-R	CTCAAAGGGAAACCGCTCA
PDCoV-N-F	CTATGAGCCACCCACCAA
PDCoV-N-R	TCCCACTCCCAATCCTGT
β-actin-F	CTTCCTGGGCATGGAGTCC
β-actin-R	GGCGCGATGATCTTGATCTTC
